# Supplementary material for: Atomic-like charge qubit in a carbon nanotube enabling electric and magnetic field nano-sensing
Source: Nat Commun. 2020 May 8;11:2299. doi: 10.1038/s41467-020-16001-5 (PMC7210973; doi:10.1038/s41467-020-16001-5)
Supplement: Supplementary file 1 — Supplementary Information [file 41467_2020_16001_MOESM1_ESM.pdf]

Supplementary Information

Atomic-like Charge Qubit in a Carbon Nanotube Enabling Electric and Magnetic Field Nano-Sensing

I. Khivrich, S. Ilani

Contents

Supplementary Note 1: Full set-up description 2

Supplementary Note 2: Gate-based charge density imaging........ 2

1. General approach........ 2
2. Imaging charging lines........ 3
3. Imaging BD transition line........ 4

Supplementary Note 3: Non-local sensitivity of charge distribution measurement by gate perturbation 4

Supplementary Note 4: Determining the qubit charge redistribution 4

Supplementary Note 5: Theoretical model and description of decoherence rate 5

Supplementary Note 6: Full calculation: self-consistent occupation and observed current . 6

Supplementary Note 7: Calibrating lever arm constants........ 8

Supplementary Note 8: Determining the qubit's potential sensitivity 8

Supplementary Note 9: Theoretical limits on the potential sensitivity 9

Supplementary Note 10: Estimating magnetic field stability........ 11

Supplementary Note 11: Measurement noise........ 11

Supplementary Note 12: Bright-dark transition lines for a range of $B||$........ 12

Supplementary Note 13: LZS simulation........ 13

Supplementary Note 14: Applying the technique for scanning probe experiments 13

Supplementary Note 1: Full set-up description

The experiment is performed in a cryogen-free dilution refrigerator. The gate voltages $V_1..V_7$ are controlled by independent DC sources. In addition, fast pulses are introduced on gates 3,4,5 using a Tabor 2184 AWG through bias T connections at the cryogenic stage.

The experiment requires highly sensitive measurement of conductance through the device. However, commonly used acoustic frequency lock-in measurements are contaminated due to mechanical vibration of the fridge through triboelectric effect in the cables[1]. In order to avoid this issue, we measure the conductance through the nanotube G at a higher (~1.45MHz) frequency using the scheme detailed in Supplementary Figure 1.

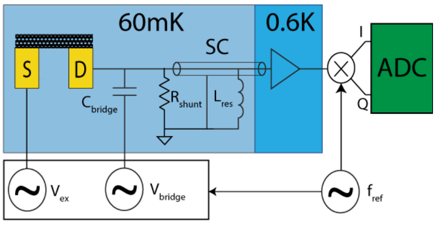

Supplementary Figure 1: Conductance measurement method The measurement is performed at $f\sim1.45MHz$ defined by the resonance frequency of an LC circuit formed by an inductor (L_{res}) and the capacitance of a superconducting cable (SC) through a custom-built two-stage HEMT amplifier. The excitation signal V_{ex} is applied on the source electrode of the device, while a separately controlled compensating signal V_{bridge} is applied on the drain in order to null direct capacitive crosstalk. The measurement result is demodulated to near baseband and sampled (ADC).

Supplementary Note 2: Gate-based charge density imaging

1. General approach

The gate-based imaging experiments described in Fig 3c, 3d and 4e in the main text give a spatial picture of how the local density along the nanotube changes as we cross transitions between the three relevant states in our experiment: $|N\rangle$, $|B\rangle$ and $|D\rangle$. These states have well defined spatial charge density distributions: $\rho_N(x), \rho_B(x), \rho_D(x)$, and across the different transition lines we measure the difference of the corresponding densities. For example across the $|N\rangle \leftrightarrow |B\rangle$ transition our experiment images $\rho_B(x) - \rho_N(x)$. Below we explain why this is the result obtained from the capacitance measurements to individual gates as is shown in the main text. The spatial potential induced along the nanotube by biasing gate i by ΔV, $\phi_i(x)$, can be calculated using electrostatic simulations, which as we have shown in past experiments[2,3] describe quantitatively well the measured potentials. This extra potential will lead to an energy shift of the coulomb peak (in the case of the $|N\rangle \leftrightarrow |B\rangle$ and $|N\rangle \leftrightarrow |D\rangle$ transitions) or to and energy shift of the qubit transition (on the $|D\rangle \leftrightarrow |B\rangle$ line) which is directly proportional to the local density above the gate $\delta \mathrm{E}_i^\beta = \int dx\, \rho_\beta(x)\phi_i(x)$, $\beta = |N\rangle, |B\rangle, |D\rangle$. The crossing between states β_1,β_2, will thus shift in energy by $\delta \mathrm{E}_i^{\beta_1,\beta_2} = \delta \mathrm{E}_i^{\beta_1}$-$\delta \mathrm{E}_i^{\beta_2}$. In the experiment we measure the the global voltage shift $\delta \mathrm{V}_i^{\beta_1,\beta_2}$ that nulls the energy perturbation introduced by changing the voltage on a single gate i by ΔV, where the global voltage is applied on a subset of the gates defined by the vector $v = (1,1,1,1,1,1,1)$ for the measurements across the Coulomb transitions and $v = (0,0,1,1,1,0,0)$ for measurements across the qubit transition, which are done only with the three central gates. We thus obtain,

$$\delta V_i^{\beta_1,\beta_2} \sum_j v_j \left(\delta E_j^{\beta_1} - \delta E_j^{\beta_2}\right) = \Delta V \left(\delta E_i^{\beta_1} - \delta E_i^{\beta_2}\right) \quad (1)$$

I.e.,

$$\rho_{\beta_1}(x_i) - \rho_{\beta_2}(x_i) = \frac{\delta V_i^{\beta_1,\beta_2}}{\Delta V} N \quad (2)$$

Where N is a normalization constant independent of i.

2. Imaging charging lines

The shifts along the global voltage axis of the bright charging line (δV_i^{NB}) are found as the shift of the center of the Coulomb blockade peak ($|N\rangle \leftrightarrow |B\rangle$) for each perturbed gate, i.e. global voltage shifts required to return the charging condition (Supplementary Fig. 2a,b). In order to find the global voltage shifts for the dark transition line, we derive its location by finding both the shift of the Coulomb blockade peak (δV_i^{NB}), and the shift of the boundary between N, D states along the magnetic field axis (ΔB_i). The latter is found by following the decrease of conductance at constant V_G, and estimating the crossing point using a linear fit. The resulting point in ($V_G, B_{||}$) is taken as the upper corner of the dark charging line (white dashed line in Supplementary Fig. 2,a). The shifts of this line in the vicinity of the upper corner are insensitive to the shifts of the lower corner, and can be estimated as $\delta V_i^{ND} = \delta V_i^{NB} + \Delta B_i \alpha$, where α is the measured unperturbed slope of the dark charging line (slope of blue line in Fig. 2c). We checked that this method of following the dark charging line (dashed white line in Supplementary Fig. 2) gives identical results to finding the position of this line by following the line connecting the two bright lines endpoints that this dark line should connect.

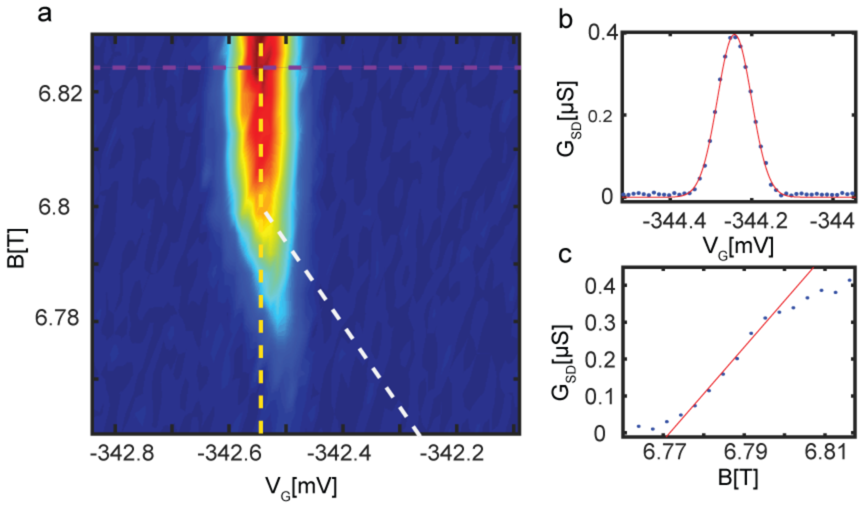

Supplementary Figure 2: Imaging charge density changes on transition lines The location of the Coulomb blockade on V_G (a, yellow line) is found by Gaussian fitting the transport at a higher B (b). The triple point where N, B, D are degenerate is located as middle point of conductance along the yellow line from a linear fit to the slope (c). The calculated N-D boundary (white line) is drawn from the triple point at a constant slope.

For a charging line (i.e., steady state conductance measurement), we use as the global gate voltage axis a vector of the form $v = (1,1,1,1,1,1,1)$. Since for a charging line, $\int (\rho_B(x) - \rho_N(x)) = 1|q_e|$, and for the values of ϕ_i obtained in using finite element simulation, $\sum \phi_i(x)$

is flat over most of the length of the nanotube (blue curve in Supplementary Fig. 4a), and N is roughly independent on ρ. As a result, we use the (Supplementary Note 2) with proper normalization to produce an estimate of $\rho_A(x_i) - \rho_B(x_i) \sim \frac{\delta V_i^{AB} q_e}{\Delta \mathrm{Vd}}$ where d is the distance between the gates. This estimate ignores the significant crosstalk between the neighboring gates, and a more correct estimation, assuming a concrete model for charge distribution, is explained in Supplementary Note 3.

3. Imaging BD transition line

Two key differences compared to the previous case are a different assumed normalization ($\int dx(\rho_{NB} - \rho_{ND}) = 0$), and a different compensation vector: $v = (0,0,1,1,1,0,0)$ due to AWG outputs not being connected to all the gates.

The shift in the location of BD transition line after perturbation δV_i^{BD} is measured as a sum of the two contributions: the shift in the Coulomb blockade peak used for readout, and the shift of the BD line with respect to the readout point. The normalization parameter, $N = \sum v_j \left(\delta E_j^B - \delta E_j^D\right)$, is estimated by imaging the two charging lines (Supplementary Note 2.2)

Supplementary Note 3: Non-local sensitivity of charge distribution measurement by gate perturbation

The charge density estimates presented in the main text (Fig. 3c,d, Fig. 4e) show the dependence of energy shift of the state on the perturbed gate number. Interpreting the resulting curve as a charge density distribution requires an additional assumption: the potential near a test charge localized above a specific gate needs to be determined by that gate only. I.e., the cross-capacitances, shown in dash in Supplementary Fig. 3 need to be much smaller than the direct capacitance (solid line). This is an approximation, justified by the geometry of the device, in which the inter-gate distance (~140 nm) is significantly larger than the height of the device above the gates (~60nm). Importantly, it allows us to draw quantitative conclusions from the measurements without relying on additional assumptions. However, this approximation is not exact, and in Supplementary Note 4 we show one possible way to better estimate the charge density distributions by introducing a concrete model.

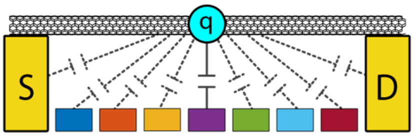

Supplementary Figure 3: Interpreting charge density imaging: due to device geometry, the gate directly under a specific section of the nanotube (labeled as a test charge q) determines the potential on it to first approximation, providing a simple interpretation of the charge density imaging results. However, a more precise interpretation can be obtained by taking into account the cross-capacitances from other gates and contacts (dashed lines).

Supplementary Note 4: Determining the qubit charge redistribution

The spatial extent of gate potentials $\phi_i(x)$ creates significant overlaps of sensitivity between different gates, resulting in smearing of the qubit charge redistribution $\rho_{BD}(x)$ measured via the capacitance to the gates (Fig 4e, main text) as compared to the actual $\rho_{BD}(x)$. We can

determine the spatial distribution of the potential produced by each one of the gates, $\phi_i(x)$, using finite element electrostatic simulations (Comsol) (Supplementary Fig. 4a). These calculated $\phi_i(x)$ were checked in our previous experiments against the actual potentials measured by scanning single electron transistor imaging, and where found to be accurate to within 10%. From Supplementary Fig. 4a, it is clear that a gate is capacitively coupled not only to the nanotube segment above it, but has also substantial coupling to positions further away along the nanotube. To deconvolve the effects of these overlaps and obtain a better estimate of $\rho_{BD}(x)$, we assume a simple form for $\rho_{BD}(x)$ that captures the essential shape of the charge redistribution: $\rho_{BD}(x) = A_1 \exp\left(-\left(\frac{x}{w_1}\right)^2\right) + A_2 \exp\left(-\left(\frac{x}{w_2}\right)^2\right)$. By optimizing the parameters A_1, A_2, w_1, w_2, we reproduce the measured gate induced shifts, $\delta V_i = \int \rho_{BD}(x)\phi_i(x)dx$. A correspondence is observed between the reproduced δV_i for the optimal values of the parameters (+, Supplementary Fig. 4b) and the measured values, shown using confidence intervals in Fig. 4b. As expected, the reconstructed charge density distribution (blue, Supplementary Fig. 4c) is narrower than the curve drawn by the measured δV_i (green, Supplementary Fig. 4c), since the latter includes the smearing by the cross capacitances of each gate to adjacent locations along the nanotube. The reconstructed shape, which represent more accurately the qubit charge density redistribution, has a $FWHM{\sim}100nm$. The overall charge change in the central node ($150nm$ width, bounded by dashed lines) is $\delta q{\sim}0.1q_e$.

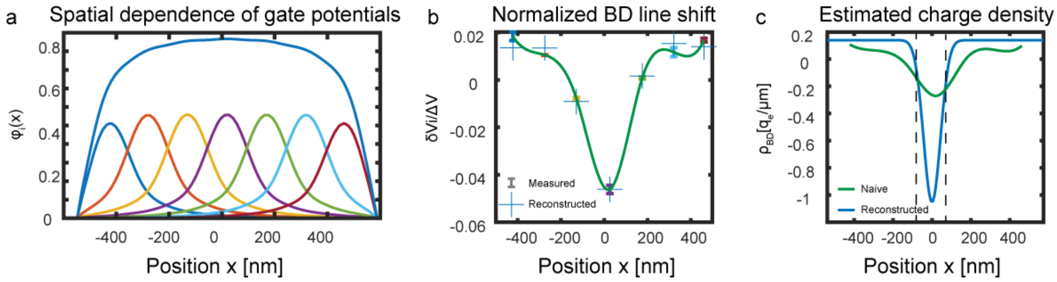

Supplementary Figure 4: Reconstructing charge density change on BD transition (a) Calculated potentials induced by the gates, normalized to voltage applied on the gates. The overlap between the adjacent gates is significant, and the naïve estimation of charge distribution using a single gate is quantitatively imprecise. We model the density change as a sum of two Gaussians, and fit their parameters so that the modelled values obtained by smearing using the gate potentials reproduces the measured values (+ signs and error bar intervals in b). The resulting reconstructed distribution (blue, c) is indeed significantly compressed as compared to naïve calculation of the density (green, c), allowing a more precise estimation of sensitivity for optimally located sources.

Supplementary Note 5: Theoretical model and description of decoherence rate

We describe the behavior of the system in Bloch equation formalism, in the manifold of the two nearly degenerate states in the far-detuned basis ($|B\rangle, |D\rangle$). The unitary evolution is described by the Hamiltonian[4]

$$H = \frac{\epsilon(t)}{2}\sigma_z + \frac{\Delta}{2}\sigma_x \tag{3}$$

Where the detuning $\epsilon(t)$ is steered in time by fast changes of the gate voltage V_G, and the tunneling rate Δ is constant. We expect the physical mechanism responsible for small, but

finite constant Δ to be a result of a small misalignment θ between the axis of the nanotube and the local direction of the magnetic field. Since the orthogonality of the two basis states results from opposite spin values, small perpendicular component of the magnetic field will result in an off-diagonal Hamiltonian term $g\mu_B\sigma_x\theta B_{||}$.

The dominant decoherence mechanism results from noise in ϵ leading to decay of the Bloch vector to the Z axis with rate γ_2. In addition, a less significant noise in Δ leads to decay to the XY plane with rate γ_1. The steady state thermal occupation vector for each detuning ϵ is given by

$$S_{th}(\epsilon) = \left(0,0,\tanh\left(\frac{\sqrt{\epsilon^2+\Delta^2}}{2T}\right)\right)^T \tag{4}$$

Where T is the temperature of the environment. The full description of the evolution of the Bloch vector is

$$\dot{S} = M(\epsilon)S + \gamma_1 S_{th}(\epsilon) \tag{5}$$

Where

$$M(\epsilon) = \begin{pmatrix} -\gamma_2 & \epsilon & 0 \\ -\epsilon & -\gamma_2 & \Delta \\ 0 & -\Delta & -\gamma_1 \end{pmatrix} \tag{6}$$

The effective decay rate T_1 is estimated by solving the equation at steady state ($\dot{S}_x = \dot{S}_y = 0$), and obtaining $\dot{S}_z = -\frac{S_z}{T_1(\epsilon)} + const$:

$$T_1^{-1}(\epsilon) = \gamma_1 + \frac{\Delta^2}{\gamma_2 + \frac{\epsilon^2}{\gamma_2}} \tag{7}$$

Thus the expected width of the observed transition line is determined by the dephasing rate γ_2, rather than temperature, as illustrated in Fig. 5 of the manuscript.

We believe that the decoherence rate of the system may be improved by increasing the tunneling element to reach the opposite limit ($\gamma_2 < \Delta$). In this case, the qubit can be operated in a 'sweet spot' regime, where the level splitting between the states is quadratically insensitive to the detuning (and to most noise sources as a result). In a similar example of a charge qubit on a carbon nanotube, operating in this regime was seen to improve the dephasing time by more than an order of magnitude[5], and hence we believe that adding a separately controlled $B_\perp$ by using a vector magnet can benefit the coherence parameters of our system similarly.

Supplementary Note 6: Full calculation: self-consistent occupation and observed current

In a naive interpretation, the dependence of the observed mean occupation $\langle P_B\rangle$ on τ_{probe} should be an exponential decay to a thermal equilibrium value. However, this interpretation neglects the possibility of incomplete initialization of the system to its ground state $|B\rangle$ at the end of the read step, which will also depend on the detuning ΔV_G and timing parameters

$\tau_{read,init}, \tau_{probe}$. Thus, in order to quantitatively interpret the dynamical behavior of the system, we need to solve a more detailed model.

Our measurement consists of repeating two steps with different values of detuning ϵ_{probe}, ϵ_{read} for the durations τ_{probe}, $\tau_{read,init}$, hence the evolution of S over a single period of the measurement is given by

$$S' = e^{M(\epsilon_{probe})\tau_{probe}}\left(e^{M(\epsilon_{read})\tau_{read,init}}\left(S - S_{th}(\epsilon_{read})\right) + S_{th}(\epsilon_{read}) - S_{th}(\epsilon_{probe})\right) + S_{th}(\epsilon_{probe}) \quad (8)$$

At steady state, we solve for $S' = S$, obtaining the value of the Bloch vector at the beginning of the read step:

$$S_0 = \left(e^{M(\epsilon_{probe})\tau_{probe}} e^{M(\epsilon_{read})\tau_{read,init}} - I\right)^{-1} \quad (9)$$

$$= \left(e^{M(\epsilon_{probe})\tau_{probe}}\left(S_{th}(\epsilon_{read})\left(I - e^{M(\epsilon_{read})\tau_{read,init}}\right) - S_{th}(\epsilon_{probe})\right) + S_{th}(\epsilon_{probe})\right)$$

Integrating over the read step (see Supplementary Fig. 5), we get the reduction of the measured conductivity $\langle G \rangle$ with respect to transport value G_0:

$$m := \frac{\langle G \rangle}{G_0} = 1 - \frac{\left(1 - S_{0,z}\right)}{2\gamma_1 \tau_{read}}\left(1 - \exp(-\gamma_1 \tau_{read})\right) \quad (10)$$

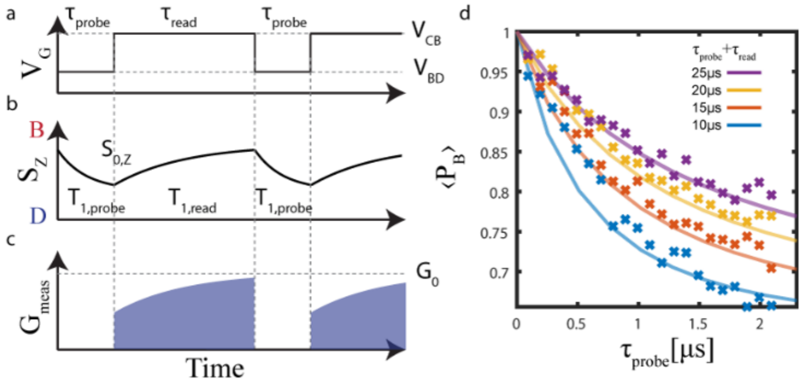

Supplementary Figure 5: accurate interpretation of the observed conductance During the read period (a), the occupation (b) is initialized towards ground state B (low S_z), while during the probe period, the probability of excited state D can increase (S_z decreases). The current through the system is only observed during the read period (c), and its value represents the average occupation of B during this period (shaded curves). The resulting rate equations explain the observed conductance at $\Delta V_G = 0$ for a range of $\tau_{read,init}, \tau_{probe}$ (d).

Substituting (4), (6), (9) into (10), we obtain an expression for the observed change in the conductance $\langle G \rangle / G_0$ as a function of three free parameters $\gamma_1, \gamma_2, \Delta$, while the electron temperature T and the conversion ratio of ΔV_G to energy scale (ϵ) are found in independent measurements. By simultaneously fitting the model to the experimental data displayed in Fig. 5b,c and Supplementary Fig. 5d (continuous lines) we obtain the values $\gamma_1 = 2\pi \times (1.5 \pm 0.15) kHz$, $\gamma_2 = 2\pi \times (185 \pm 6) MHz$, $\Delta = 2\pi \times (2 \pm 0.12) MHz$, where the confidence intervals are 2σ and are obtained using Monte Carlo approach.

To obtain a more useful formula, an approximate solution of the same problem can be found by looking at the evolution of z component of the Bloch vector. Two effective values of the decay rate (7) are used: $T_{1,read}^{-1} = T_1^{-1}(\epsilon_{read})$, $T_{1,probe}^{-1} = T_1^{-1}(\epsilon_{probe})$. In this case, the self-consistent relation (9) can be written as

$$S_{0,z} = \frac{S_{th,z}\left(e^{\frac{\tau_{probe}}{T_{1,probe}}} - 1\right)}{e^{\frac{\tau_{probe}}{T_{1,probe}}} - e^{-\frac{\tau_{read,init}}{T_{1,read}}}} \tag{11}$$

Supplementary Note 7: Calibrating lever arm constants

The measurement is performed in terms of the detuning ϵ between the states B, D, and in order to obtain the sensitivity of electric and magnetic field measurements, we need to calibrate the electrostatic lever arm factors $\alpha_B, \alpha_D = q_e^{-1}\partial E_{B,D}/\partial V_G$ where δV is a voltage of interest (in the following discussion, common voltage on three central gates 3,4,5), and the magnetic moments $\mu_B, \mu_D = \partial E_{B,D}/\partial B_{||}$. Using the measured slopes of red and blue lines in Fig. 2c ($S_B = \mu_B/\alpha_B$, $S_D = \mu_D/\alpha_D$), and the slope of the interface line in Fig. 4d $K = (\mu_B - \mu_D)/(\alpha_B - \alpha_D)$, we obtain

$$\frac{\alpha_D}{\alpha_B} = \frac{1 - KS_B}{1 - KS_D} \tag{12}$$

Using the value of $\alpha_B \sim 0.15$ measured directly from Coulomb diamond diagram (Supplementary Fig. 6), we obtain $\alpha_D \sim 0.2$, $\mu_B \sim -0.08 meV/T$, $\mu_D \sim 1.1 meV/T$ for the states discussed in the manuscript.

The obtained lever arm factor for the qubit transition $\alpha_D - \alpha_B \sim 0.05$ is consistent with the lever arm factor independently obtained from the spacing between LZS interference fringes:

$$\alpha = \frac{2\pi\hbar f_{LZS}}{55\mu V q_e} \sim 0.0525 \tag{13}$$

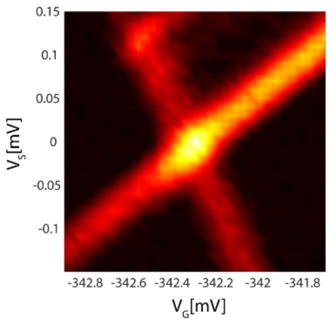

Supplementary Figure 6: Calibrating lever arm constants The lever arm factor $\alpha_B = \partial E_B/\partial V_G$ is extracted from the Coulomb diamond measurement. Combined with the measurements in Fig. 1f and Fig. 3d, full set of values of lever arm factors $\alpha_B, \alpha_D, \mu_B, \mu_D$ is found.

Supplementary Note 8: Determining the qubit's potential sensitivity

We determine the qubit's sensitivity to electrical potential using the data displayed in Supplementary Fig. 7. In order to estimate the measurement noise σ_G obtained in practice, data from 6 consecutive runs in a flat region of the measurement is taken (black box). By plotting the envelope calculated as mean of the measurement $\pm 1.5\sigma_G$, we verify that the samples are uniformly confined by this envelope, and the obtained noise value can be used

for all values of ΔV_G. Using σ_G, the slope (dashed line) and the lever arm factor (Supplementary Note 6), we find the sensitivity to detuning to be $\delta\epsilon = 60neV/\sqrt{Hz}$.

The shift of detuning due to a local electrical potential $\phi(x)$ is given by $\delta\epsilon = \int \phi(x)\,\rho_{BD}(x)$. As is apparent from Supplementary Fig. 4c, the qubit charge redistributes from the center to the sides ($\rho_{BD}(x)$ is negative in a region of width W~$100nm$ in the center and positive on the sides). This means that the qubit will be insensitive to potentials whose wavelengths are much larger than W, making it immune against far field potential noise. The optimal sensitivity in potential measurements will be obtained when the measured feature is comparable in size to the resolution that is set by W. In this case the potential sensitivity can be determined to be $\delta V = \frac{\delta\epsilon}{\delta q} \sim 600nV/\sqrt{Hz}$, where δq is the redistributed charge (Supplementary Note 4). Note that the sensitivity to voltage on gate 4, $\delta V_4 \sim 1.4\mu V$ is about two times worse as the potential that this gate produces, $\phi_4(x)$, is smeared on a scale larger than W.

Supplementary Note 9: Theoretical limits on the potential sensitivity

The potential sensitivity obtained in the experiment (Supplementary Note 7), although extremely good, can still be improved if few limiting factors in the current experiment are improved. To get a sense of what is the optimal performance expected for this qubit based on its measured properties, we analyze its theoretical sensitivity limits below:

The measured quantity in our experiment is the dark vs. bright state occupation near the qubit transition. This quantity depends very sharply on the detuning, ϵ, between these states. The detuning can be modified by a modulation of the magnetic field ($\delta\epsilon = (\mu_B - \mu_D)\delta B_{||}$) where μ_B, μ_D are the magnetic moments of the two states, or by electric potential ($\delta\epsilon = (\alpha_B - \alpha_D)\delta V$), where α_B, α_D are the lever arm factors of the gate capacitance to the charge distributions of the states. The detuning is detected through the modulation of current $\delta I = I_0 m(\delta\epsilon)$ where I_0 is the readout current measured on Coulomb blockade peak. The sensitivity of m to ϵ can be seen from Supplementary Notes 7, 11 to be of the form $\frac{\partial m}{\partial \epsilon} \sim A\hbar\gamma_2$, where A is a numerical factor depending on the timing parameters and approaching the value of ~ 10 when optimized. The signal to noise ratio for measuring m, SNR_m in our case is limited by our contact resistance, $R_{contact}$. In such a case of resistive contacts, SNR_m is determined by the amplifier noise floor $i_n \sim 25fA/\sqrt{Hz}$ ($SNR_m = \frac{I_o}{i_n}$, where I_0 is limited by the maximal AC excitation for conductance measurement $V_{ex} \sim 2.3k_BT/q_e$ and the device contact resistance $R_{contact}$). In total, for this case, the sensitivity to detuning between the energies of B,D is:

$$\delta\epsilon_{contact\ resistance\ limited} \sim \frac{4\hbar\gamma_2 i_n q_e R_{contact}}{k_B T} \tag{14}$$

For vertex properties described in the manuscript, $\delta\epsilon \sim 45neV/\sqrt{Hz}$, which is translated to $\delta V \sim 450nV/\sqrt{Hz}$.

The device in this study had a far from optimal contact resistance, $R_{contact} \sim 2M\Omega$. In principle this resistance could be improved down to tens of $k\Omega$ range, improving the sensitivity of the described method. Ultimately, the signal to noise of measuring m will be limited by the shot

noise of the measurement and will become insensitive to $R_{contact}$ (since the current during the readout stage assumes one of two possible values): $SNR_m = 1/\sqrt{\tau_{read,init} + \tau_{probe}}$. For a finite bandwidth BW for setting the gate voltages determining the minimal $\tau_{probe} \sim BW^{-1}$, the optimal sensitivity is obtained for $\tau_{read} \sim \tau_{probe} T_1^{probe} / T_1^{read}$, hence the optimal value is $SNR_m = \frac{\sqrt{\gamma_1 \gamma_2 BW}}{\Delta}$. Thus, the ultimate bound for sensitivity is

$$\delta\epsilon_{shot\ noise\ limited} \sim \frac{10\sqrt{\gamma_2}\hbar\Delta}{\sqrt{\gamma_1}\sqrt{BW}} \tag{15}$$

For the device equivalent to those presented in the manuscript and $BW \sim 1GHz$ $\delta\epsilon_{shot\ noise\ limited} \sim 4neV/\sqrt{Hz}$, which translates to $\delta V \sim 40nV/\sqrt{Hz}$ and $\delta B \sim 3.5\mu T/\sqrt{Hz}$.

The factors leading to the difference between the naive estimation of the expected performance (Supplementary Note 6) and the obtained result include slow drifts of device conductance, which can be avoided performing duty cycle measurements. In addition, since the device is sensitive to both magnetic and electric field, slow drifts in local electric field will contribute to measurement noise of magnetic field and vice versa. The device can be optimized for a required type of measurement by choosing other working points in (V_G, B) space, which may have different coupling differences $(\alpha_B - \alpha_D)$, $(\mu_B - \mu_D)$. For instance, in Supplementary Fig. 7b we choose a vertex with significantly smaller $\alpha_B - \alpha_D$, improving the resulting magnetic field sensitivity to $39\mu T/\sqrt{Hz}$ (evaluated in the same way). Our sensing relies on the coherence limited transition of the qubit, and in this sense is similar to relaxometry measurements in NV centers, used to probe high frequency noise. So far we could not use the full potential of coherent manipulation of the qubit, due to the rather short T_2^*. With improvement in T_2^* it should be possible to use dynamic decoupling protocols, and thus be limited by T_2 that is likely to be significantly longer. In this case further improvement to the sub nV potential sensitivity might become possible, as was demonstrated in singlet-triplet qubits in GaAs double quantum dots[6].

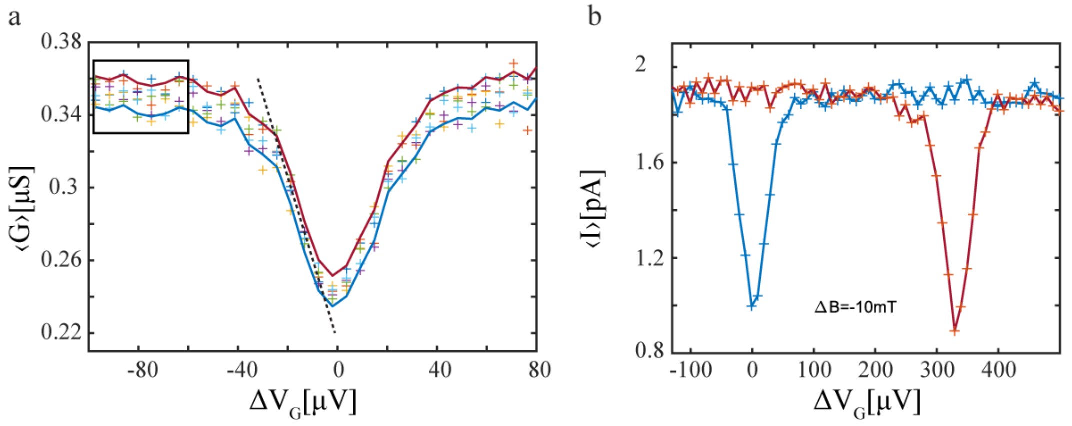

Supplementary Figure 7: Estimating detection sensitivity a. Noise statistics are estimated from 6 consecutive scans (+); noise power σ_G extracted in a far-detuned region (black box) is seen to be uniformly correct for all detuning values from the envelope curves, separated by $3\sigma_G$. The sensitivity to ΔV_G is obtained from σ_G and the maximal slope of the conduction dip (dashed line) b. Choosing a vertex with smaller difference between the charge distributions of $|B\rangle, |D\rangle$ leads to broadening of the conduction dip and increase in $B_{||}$-dependence of its location, shown in two graphs for slightly different magnetic field values. Overall, the sensitivity to $B_{||}$ is improved.

Supplementary Note 10: Estimating magnetic field stability

The superconducting magnet in the experiment could not be used in persistent current mode, and the field was regulated using a closed loop controller. As a result, $B_{||}$ in the experiment fluctuates, limiting the obtainable measurement sensitivities and coherence times. To estimate the magnetic field fluctuations, we recoded the field values reported by the controller (Supplementary Fig. 8), from which we estimate magnetic field stability of $\sim 25\ \mu\mathrm{T}\ rms$, translating to maximal observable dephasing time $T_2^\star \sim 15\ \mathrm{ns}$. Note that these measurements show only the low frequency components of the magnetic fluctuations, and other faster fluctuations might be also present.

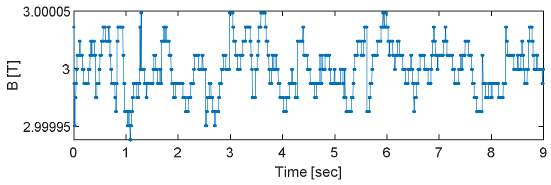

Supplementary Figure 8: Measured fluctuations of the magnetic field The magnetic field reported by the closed loop controller driving the superconducting coil are plotted for setpoint B=3T.

Supplementary Note 11: Measurement noise

The noise spectrum of the measured signal, as a function of the detuning ΔV_G is shown in Supplementary Fig. 9. In this experiment, V_{bridge} (See Supplementary Fig. 1) is balanced as to obtain zero signal in the far-detuned regime ($|\Delta V_G| \gg 0$). The displayed spectrum is centered around the demodulation frequency ($f_{LO} = 1.46\ \mathrm{MHz}$), which is chosen to be close to the peak of the response of the measurement LC tank (Supplementary Fig. 1). On the far-detuned graphs (red, blue), we observe noise envelope resulting from the shape of LC tank response. In addition, we observe spurious lines at $\sim 5\ \mathrm{kHz}$, $\sim 15\ \mathrm{kHz}$ (black arrows) resulting from high harmonics of the fast gating sequence periodicity ($\tau_{period} = \tau_{read} + \tau_{probe}$), and at 0 Hz (blue arrow) resulting from leakage and DC offset drifts. The excitation frequency is $f_{ex} \sim f_{LO} + 9.5\ \mathrm{kHz}$ (red arrow), and the difference between the three curves is only observed near this frequency, as an harmonic signal (due to different conductance) for the zero detuning point (yellow), and a wide skirt resulting from shot noise of the conductance (due to random outcome in each repetition of the measurement sequence). This effect is observed for long measurement period τ_{period} (50 µs for these measurements), decreasing for shorter period times. Overall, for short measurement period times, the noise floor is detuning independent for all frequencies, and the performance is limited by the conductance measurement sensitivity, as seen in Supplementary Fig. 7.

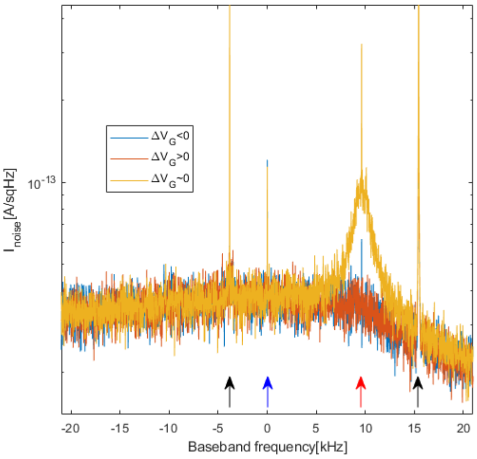

Supplementary Figure 9: Measurement noise in current experiment The regime of constraints determining the potential sensitivity of the device (Supplementary Note 9) in the current experiment is found by measuring the noise spectrum as a function of ΔV_G in a time domain measurement, shown here for far detuned (blue, red), and zero detuning (yellow) cases. Black and blue arrows mark spurious signals resulting from the measurement technique, while the red arrow marks the frequency at which the conductance through the device is measured. As the observed noise floor of the measurement does not depend on the detuning, we conclude that the practical sensitivity of the device is currently limited by the sensitivity of conductance measurement.

This clearly shows that there is still a place for improving the sensitivity: by decreasing the resistance of the contacts to the nanotube, the transconductance of the device will be improved. In this way we will obtain better voltage/charge sensitivity, which would be ultimately limited by the charge noise of the system, a limit that was not reached in the current measurements due to the large contact resistance.

Supplementary Note 12: Bright-dark transition lines for a range of $B_{||}$

In order to implement the demonstrated measurement scheme, the control parameters of the system, V_G and $B_{||}$ need to be tuned to obtain a dark state with sufficiently long lifetime, sufficiently close to a Coulomb blockade peak to allow fast gating transitions between readout (B-N) and measurement (B-D) lines. We demonstrate that these requirements can be fulfilled over a range of values of $B_{||}$ by showing a number of observed B-D transition lines (dashed green lines in Supplementary Fig. 10), shown with respect to conductance measurement of the device (electrons side). The maximal distances of the displayed lines from the Coulomb peaks represent the maximal reachable fast gate voltage transition amplitude in our set-up. The observed detuning dependence of the transition lines (blue) is shown for each case. Overall, while only some of the vertices in the transport diagram were measured, the displayed lines allow replicating the experiment over a significant fraction of $B_{||} = 3T..6T$ range. In additional measurements we have also observed the effect in other parts of the $(V_G, B_{||})$ diagram, for both electron and hole doped dots, and have seen that similar qubit transition exists at least up to $B_{||} = 8T$ (not shown). The additional lines have similar sharpness and dependence on magnetic field, and hence can also be used to form a local sensor, achieving similar values of sensitivity.

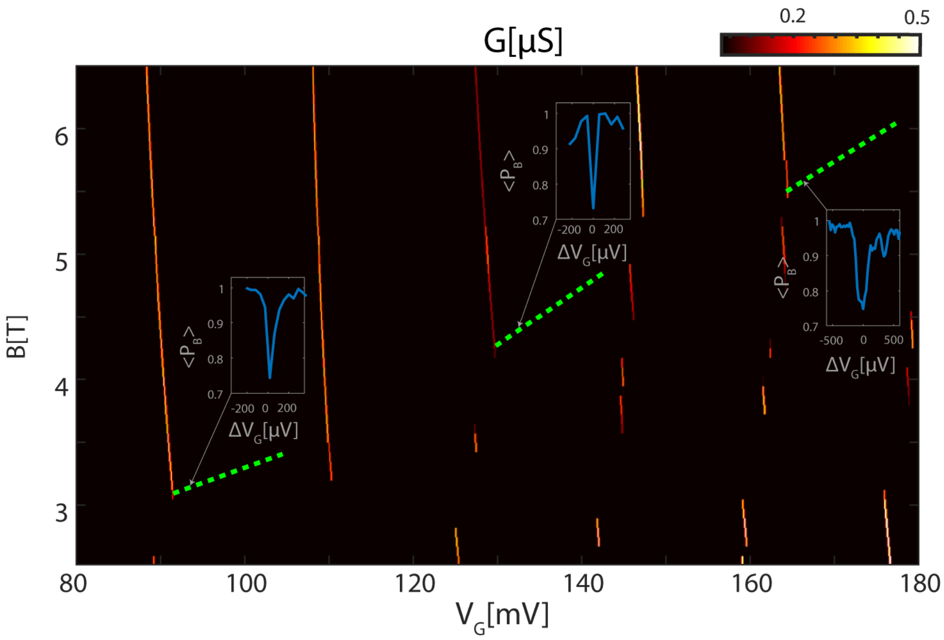

Supplementary Figure 10: bright-dark transition lines for a range of $B_{||}$ Zig-zag pattern of Coulomb blockade(orange colormap). Measured locations and slopes of lines of transition to dark state obtained using the time-domain method shown in green dashed lines, terminating at distance from CB peak limited by maximal fast gating jump amplitude (setup - limited). The insets show the observed $\langle P_B\rangle(\Delta V_G)$ (equivalent to Fig. 4c). In some cases, these lineshapes are more complex than the simple case described in the manuscript, and indicate the existence of other states in addition to N,B,D. Despite this complexity, the observed magnetic moments and line sharpness are similar to the simple qubit described in the paper, thus allowing similar measurement sensitivity. Overall, using the displayed lines, the electric and magnetic sensing mechanism can be used for most of the values of $B_{||}$ between 3 T and 6 T (We have measured similar lines up to 8 T, not shown).

Supplementary Note 13: LZS simulation

The simulation is done using QuTip package[7]. We simulate a system described by Hamiltonian (Supplementary Note 3) where $\epsilon(t)$ is modulated harmonically, and the decoherence operators $\sqrt{\frac{\gamma_1}{2}}\sigma_-$ and $\sqrt{\frac{\gamma_2}{2}}\sigma_z$. While a qualitatively similar result can be obtained by finding the steady state $\langle P_B\rangle$ of the resulting Lindblad equation, a better quantitative match is obtained by calculating the steady state occupation for two consecutive propagation steps, in which $\epsilon(t) = \epsilon_0 + A_{LZS}\sin(\omega t)$ for time τ_{probe}, and $\epsilon(t) = \epsilon_{CB}$ for time $\tau_{read,init}$.

Supplementary Note 14: Applying the technique for scanning probe experiments

In order to implement this measurement technique as a scanning nano-probe, the experimental set-up will need to include the following elements:

1) High frequency cabling to the microscope. While not trivial, cryogenic high-frequency cabling was implemented in several scanning setups (e.g. NV centers, scanning microwave probes, etc.). High frequency coaxial cables can exert forces on scanning piezo stages. We note, however, that high-bandwidth signals need to be applied only to the detector, and not to the sample. Thus, the problem is avoided if the sample is mounted on the piezo stage, and the probe cantilever is fixed.
2) Vector magnet - the qubit requires magnetic field parallel to the nanotube axis. Ideally, we would like to also have an independent control over the perpendicular

field. This field is often the one that most strongly controls the physics of a 2D sample under study. This independent control of fields would be best achieved using a vector magnet.

The process of tuning the device is more complex than using the same device as a SET. In addition to locating Coulomb blockade peaks, we need to trace their positions as a function of $B_{||}$, and locate the transitions between different wavefunctions, as sharp changes in visibility of Coulomb blockade conduction lines (see Supplementary Fig. 10). For each switching point in (V_G, B), we check whether a slow tunneling rate between the two switching states is observed, indicating a spin flip. This is done by performing the time domain sequence in Fig. 4a to observe whether sharp lines of decreased conductance are observed (Fig. 4c). In the device discussed in this work, roughly 1/4 of the switching points exhibited sharp transition lines, over multiple cooldowns which influenced the transport characteristics of the device. While overall the process is time consuming, it needs to be done only once to allow calibrating the device as a sensor, and thus will not be a limiting factor for scanning measurements with the qubit.

In order to choose a suitable carbon nanotube to demonstrate the effect, four properties of the chosen state are required:

1. It should have two basis states that are roughly orthogonal, namely the tunnelling element between them should be small.
3) These states should have a large difference in their tunneling rates to both leads (dark/bright state). This is crucial for the simple transport readout that we showed in the paper.
4) The states should have significantly different charge distributions to allow electric field detection.
5) The states should have different magnetic moments to allow magnetic field detection.

Current measurements demonstrate these properties independently, without relying on a specific model, for a range of regimes (electron/hole doped dot, range of occupation numbers and magnetic fields). We note that similar systematic dark-bright behaviour was observed in measurements that we performed previously (see ref. 29). We expect the effect to be quite universal for a variety of near-metallic carbon nanotube devices.

Supplementary References

1. Kalra, R. *et al.* Vibration-induced electrical noise in a cryogen-free dilution refrigerator: Characterization, mitigation, and impact on qubit coherence. *Rev. Sci. Instrum.* **87**, 073905 (2016).
2. Waissman, J. *et al.* Realization of pristine and locally tunable one-dimensional electron systems in carbon nanotubes. *Nat. Nanotechnol.* **8**, 569–574 (2013).
3. Shapir, I. *et al.* Imaging the electronic Wigner crystal in one dimension. *Science* **364**, 870–875 (2019).
4. Shevchenko, S. N., Ashhab, S. & Nori, F. Landau-Zener-Stückelberg interferometry. *Phys. Rep.* **492**, 1–30 (2010).
5. Penfold-Fitch, Z. V., Sfigakis, F. & Buitelaar, M. R. Microwave Spectroscopy of a Carbon Nanotube Charge Qubit. *Phys. Rev. Appl.* **7**, 054017 (2017).
6. Dial, O. E. *et al.* Charge Noise Spectroscopy Using Coherent Exchange Oscillations in a Singlet-Triplet Qubit. *Phys. Rev. Lett.* **110**, 146804 (2013).
7. Johansson, J. R., Nation, P. D. & Nori, F. QuTiP 2: A Python framework for the dynamics of open quantum systems. *Comput. Phys. Commun.* **184**, 1234–1240 (2013).
